# Cost-effectiveness analysis of umeclidinium bromide/vilanterol 62.5/25 mcg versus tiotropium/olodaterol 5/5 mcg in symptomatic patients with chronic obstructive pulmonary disease: a Spanish National Healthcare System perspective

**DOI:** 10.1186/s12931-018-0916-7

**Published:** 2018-11-20

**Authors:** M. T. Driessen, J. Whalen, B. Seewoodharry Buguth, L. A. Vallejo-Aparicio, I. P. Naya, Y. Asukai, B. Alcázar-Navarrete, M. Miravitlles, F. García-Río, N. A. Risebrough

**Affiliations:** 1 Value Evidence and Outcomes, GSK, 980 Great West Road, Brentford, Middlesex TW8 9GS UK; 2 ICON Health Economics, ICON plc, Abingdon, UK; 3 Departamento de Evaluación de Medicamentos, GSK, Tres Cantos, Madrid, Spain; 4 Global Respiratory Franchise, GSK, Brentford, Middlesex, UK; 5 Hospital de Alta Resolución de Loja, Granada, Spain; 6 Pneumology Department, Hospital Universitari Vall d’Hebron, Barcelona, Spain; 7 Hospital Universitario La Paz-IdiPAZ, Madrid, Spain; 8 ICON Health Economics, ICON, Toronto, ON Canada

**Keywords:** Bronchodilators, Chronic obstructive pulmonary disease, Cost effectiveness, Economic evaluation, Health resources, LAMA/LABA, National Healthcare System perspective, QALY, Spain, Utility

## Abstract

**Background:**

A head-to-head study demonstrated the superiority of once-daily umeclidinium bromide/vilanterol (UMEC/VI) 62.5/25 mcg on trough forced expiratory volume in 1 s (FEV_1_) versus once-daily tiotropium/olodaterol (TIO/OLO) 5/5 mcg in symptomatic patients with chronic obstructive pulmonary disease (COPD). This analysis evaluated the cost effectiveness of UMEC/VI versus TIO/OLO from a Spanish National Healthcare System perspective, using data from this study and Spanish literature.

**Methods:**

This analysis was conducted from the perspective of the Spanish National Healthcare System with a 3-year horizon as base case. A disease progression model using a linked risk equation approach was used to estimate disease progression and associated healthcare costs, and quality-adjusted life years (QALYs). The Evaluation of COPD Longitudinally to Identify Predictive Surrogate Endpoints (ECLIPSE) study was used to develop the statistical risk equations for clinical endpoints, and costs were calculated using a health state approach (by dyspnea severity). Utilities for QALY calculation were estimated using patient baseline characteristics within a regression fit to Spanish observational data. Treatment effect, expressed as change from baseline in FEV_1_ was obtained from the head-to-head study and used in the model (UMEC/VI minus TIO/OLO difference: + 52 mL [95% confidence interval: 28, 77]). Baseline patient characteristics were sourced from Spanish literature or the head-to-head study if unavailable. A scenario analysis using only the intent-to-treat (ITT) population from the head-to-head study, and sensitivity analyses (including probabilistic sensitivity analyses), were conducted. Direct healthcare costs (2017 Euro) were obtained from Spanish sources and costs and benefits were discounted at 3% per annum.

**Results:**

UMEC/VI was associated with small improvements in QALYs (+ 0.029) over a 3-year time horizon, compared with TIO/OLO, alongside cost savings of €393/patient. The ITT scenario analysis and sensitivity analyses had similar results. All probabilistic simulations resulted in UMEC/VI being less costly and more effective than TIO/OLO.

**Conclusion:**

UMEC/VI dominated TIO/OLO (more effective and less expensive). These results may aid payers and decision-makers in Spain when making judgements on which long-acting muscarinic antagonist/long-acting β_2_-agonist (LAMA/LABA) treatments can be considered cost effective in Spain.

**Electronic supplementary material:**

The online version of this article (10.1186/s12931-018-0916-7) contains supplementary material, which is available to authorized users.

## Background

Chronic obstructive pulmonary disease (COPD) is a disabling respiratory disease characterized by airflow limitation and persistent breathing difficulties [1]. COPD is associated with a high clinical and economic burden worldwide, impacting the patient’s quality of life and causing significant costs, associated with clinical care [1]. In Spain, the prevalence of COPD in patients aged 40–80 years old is approximately 10.2% and prevalence is higher in men compared with women (15.1% versus 5.6%, respectively) [2]. Geographic variations in prevalence range from 6.2–16.9% [3]. The annual cost of COPD in Spain was estimated at €239 million in 1997 and €507 million in 2000 [4, 5].

Pharmacological treatment for COPD aims to improve patient symptoms and reduce the risk of exacerbation [1]. The cornerstone of pharmacological therapy for COPD is bronchodilation with a long-acting muscarinic antagonist (LAMA), a long-acting β_2_-agonist (LABA), or a LAMA/LABA combination either as initial therapy or escalation from monotherapy, depending on the severity of breathlessness and the patient’s risk of exacerbations [1, 6–8]. The Global Initiative for Chronic Obstructive Lung Disease (GOLD) 2018 report recommends dual LAMA/LABA therapy for patients initially on monotherapy who continue to experience exacerbations or patients with severe or persistent breathlessness [1].

The Spanish guidelines for pharmacological treatment of stable COPD (Guía española de la enfermedad pulmonar obstructiva crónica, GesEPOC) [9] include a number of GOLD principles and recommendations; however, GesEPOC follows an alternative suite of phenotype-based recommendations for the evaluation and treatment of COPD [9]. GesEPOC proposes four phenotypes that determine differential treatment: non-exacerbators; mixed COPD-asthma; exacerbators with emphysema; and exacerbators with chronic bronchitis [10]. Within this framework, dual LAMA/LABA therapy is recommended in non-exacerbators who remain symptomatic after bronchodilator monotherapy, and in patients with emphysema or chronic bronchitis at risk of exacerbations [10].

Various LAMA/LABA combinations are currently available in Spain. Umeclidinium/vilanterol 62.5/25 mcg (UMEC/VI) is a once-daily single inhaler LAMA/LABA therapy, approved for the treatment of COPD by the European Medicines Agency (EMA) in 2014 [11]. The safety and efficacy of UMEC/VI has been extensively investigated and studies have shown improvements in lung function with UMEC/VI compared with placebo [12, 13], UMEC or VI monotherapy [12, 14, 15], tiotropium (TIO) [14, 16, 17] and inhaled corticosteroid (ICS)/LABA combination therapy [18, 19]. TIO/olodaterol (TIO/OLO) was first approved as treatment for patients with COPD in 2015 [20]. A recent systematic literature review demonstrated that treatment with TIO/OLO provides significant improvement in lung function when compared with TIO and OLO monotherapies and when compared with ICS/LABA combination therapy [21].

Until recently, no direct comparison between once-daily LAMA/LABA combination therapies had been conducted, although indirect comparisons of double blind trials highlighted potential for efficacy differences within the LAMA/LABA treatment class in favor of UMEC/VI [22, 23]. A 12-week head-to-head study of UMEC and TIO monotherapies demonstrated that UMEC was superior to TIO in trough forced expiratory volume in 1 s (FEV_1_), with a treatment difference of 59 mL (95% confidence interval [CI]:29, 88 mL) [24], and a recent open-label, 8-week crossover head-to-head study demonstrated that this superiority was maintained when UMEC and TIO were administered as part of a LAMA/LABA dual therapy [25]. This LAMA/LABA head-to-head study, UMEC/VI 62.5/25 mcg was compared with TIO/OLO 5/5 mcg in symptomatic patients with moderate COPD who were naïve to ICS at study entry [25]. The study showed that UMEC/VI was superior to TIO/OLO in the intent-to-treat population for FEV_1_ improvement at 8 weeks with a treatment difference of 52 mL (95% CI: 28, 77 mL) [25].

UMEC/VI has already been shown to be cost effective when compared with TIO alone from a Spanish National Healthcare System perspective [26]. Following the completion of the head-to-head study comparing UMEC/VI and TIO/OLO, this study aimed to assess the cost effectiveness of UMEC/VI versus TIO/OLO from a Spanish National Healthcare System perspective [25]. Given that the annual drug acquisition costs for UMEC/VI are lower than TIO/OLO in Spain, it was anticipated that this analysis would find that UMEC/VI dominated TIO/OLO (as a more effective and less expensive treatment). However, testing this hypothesis through sensitivity analyses is important to demonstrate the robustness of this assumption and to adequately inform healthcare decision makers.

## Methods

### Objectives

A cost-effectiveness analysis was performed comparing once-daily UMEC/VI with once-daily TIO/OLO in symptomatic patients with stable COPD at low risk of exacerbations from the perspective of the Spanish National Healthcare System over a 3-year time horizon, using data from published literature and the head-to-head study of UMEC/VI versus TIO/OLO (study funded by GSK, study number 204990; NCT02799784) [25].

### Design of the clinical study included in the analysis

The UMEC/VI versus TIO/OLO head-to-head study was a randomized, 8-week, open-label, two-period crossover study in symptomatic patients with moderate COPD [25]. To minimize the potential for bias in the assessment of the primary efficacy endpoint of trough FEV_1_, given the open-label design, all spirometry assessments were performed by investigational staff blinded to treatment allocation throughout all study phases. Eligible patients were ≥ 40 years of age, with a diagnosis of COPD [27], a modified Medical Research Council (mMRC) dyspnea score of ≥2, a smoking history ≥10 pack-years, a post-bronchodilator FEV_1_/forced vital capacity ratio < 0.70 and a post-bronchodilator FEV_1_ ≤ 70% and ≥ 50% of predicted normal values.

Patients were randomized to receive UMEC/VI 62.5/25 mcg one inhalation once daily via the Ellipta dry powder inhaler for 8 weeks, followed by TIO/OLO 5/5 mcg (via two inhalations once daily of TIO/OLO 2.5/2.5 mcg) using the Respimat soft mist inhaler for 8 weeks, or vice versa, with an interim 3-week washout between each 8-week treatment period. The primary endpoint was trough FEV_1_ at week 8 in both the ITT and per-protocol (PP) population, with a non-inferiority margin and superiority margin of − 50 mL and 0 mL for the lower bound of the 95% CI in the PP and ITT populations, respectively. Other lung function and patient-reported outcomes were also assessed [25].

### Cost-effectiveness model

The GALAXY COPD disease progression model was used to perform cost-effectiveness calculations; development of the model and internal/external validation, have been previously published [28–32]. A linked risk equation approach was used within the model to estimate disease progression. Associated healthcare costs such as drug costs, hospitalization costs and outpatient visits, as well as the impact on quality-adjusted life years (QALYs) and survival were estimated based on the resulting clinical outcomes. The model assigned general costs of follow-up based on the proportion of patients with various levels of dyspnea in each model cycle. Post hoc analyses of the Evaluation of COPD Longitudinally to Identify Predictive Surrogate Endpoints (ECLIPSE; NCT00292552) [33] study was used to develop the statistical risk equations for the epidemiological framework. The model uses a 1-year cycle length.

Ethics approval was not required for the study as data for the model were derived from previously conducted studies, for which ethical approval had been obtained.

### Study perspective

The cost-effectiveness analyses were conducted from a Spanish National Healthcare System perspective and costs included direct healthcare-related costs, but not costs associated with a societal perspective (e.g. non-medical costs and indirect costs such as absenteeism and presenteeism).

### Model inputs

#### Population

Patient characteristics for the base case analysis were based on two Spanish observational studies [34, 35], in order to analyze a population that is representative of the Spanish population initiating LAMA/LABA therapy. Where data could not be found, for example for clinical characteristics, data from the UMEC/VI versus TIO/OLO head-to-head study were used [25] (Table 1). All characteristics used for the base case analysis were validated by three clinical experts from Spain (initials of clinical experts: MM, BA-N, and FG-R). Data on baseline fibrinogen concentration and baseline 6-min walk test (6MWT) distance were not available from the UMEC/VI versus TIO/OLO head-to-head study; these data were estimated using equations developed within the model using baseline data from the ECLIPSE study [33] (Table 1).

**Table 1:** Model inputs: baseline demographics by base case and ITT population, and resource costs

Parameters	Base case analysis	ITT scenario analysis
Female, %	19.2 [34]	39.8
Age (years), mean (SE)	68.2 (0.4) [34]	64.4 (0.6)
Smoking status (current smokers), %	23.1 [35]	53.0
Any cardiovascular comorbidity, %	26.3^a^	26.3^b^
Any other comorbidity, %	78.4^a^	78.4
History of exacerbation, ≥1 moderate or severe in the previous 12 months, %	18.2^a^	18.2
BMI (kg/m^2^), %		
< 21	7.1 [35]	10
21–30	60.8 [35]	50
> 30	32.1 [35]	40
mMRC score ≥ 2, %	100^a^	100
Number of moderate and severe exacerbations in previous year, mean (SE)	0.2 (0.03)^a^	0.2 (0.03)
Number of severe exacerbations in previous year as a % of total previous year exacerbations, mean	13.7^a^	13.7
Baseline FEV_1_% predicted, mean (SD)	59.6 (5.6)^a^	59.6 (5.6)
Baseline FEV_1_ mL, mean (SE)	1563 (28.6)^a^	1563 (28.6)
Height (cm), mean (SE)	167.6 (0.3) [36]	169.9 (0.6)
Fibrinogen (mcg/dL), mean	456.7^c^	453.2^c^
SGRQ score, mean (SE)	42.7 (0.3) [56]	43.1 (1.0)^d^
6MWT distance (m), mean	346.1^c^	349.9^c^
Exacerbation event costs (€)		
Moderate exacerbation	72.76	
Severe exacerbation	4466.09	
Annual disease management costs (€/year)		
Without dyspnea symptoms	524.87	
With dyspnea symptoms several days per week	699.98	
With dyspnea symptoms most days per week	925.85	

*BMI* body mass index, *FEV*_*1*_ forced expiratory volume in one second, *ITT* intent-to-treat; mMRC, modified Medical Research Council, *SD* standard deviation, *SE* standard error; *SGRQ* St. George’s respiratory questionnaire, *6MWT* 6-min walk test

^a^Spanish data not available from publications so sourced from head-to-head study [25]

^b^cardiovascular comorbidity defined as any cardiac disorder (coronary artery disease, myocardial infarction, arrhythmia, or congestive heart failure) or cerebrovascular accident

^c^predicted using GALAXY model

^d^predicted using GALAXY model SGRQ-C risk equation and converted to SGRQ

#### Efficacy input parameters

Treatment effect was measured by change from baseline in post-bronchodilator FEV_1_, using data from the UMEC/VI versus TIO/OLO head-to-head study (UMEC/VI: 180 mL; TIO/OLO: 128 mL; difference: 52 mL, 95% CI: 28, 77 mL) [25]. These absolute treatment effects (UMEC/VI: 180 mL; TIO/OLO: 128 mL) were applied to the baseline FEV_1_ and differential treatment effect (52 mL) was maintained over the time horizon as long as patients remained on therapy. The starting FEV_1_ values and subsequent improvements in FEV_1_ and other COPD disease factors built into the model at time 0 are outlined in Additional file 1.

#### Cost inputs

Total costs accounted for the drug acquisition costs, the costs of exacerbation events (moderate or severe) and the costs of follow-up according to the frequency of dyspnea symptoms. All costs were estimated in 2017 Euros and were adapted from Spanish sources.

National list prices for drug acquisition costs, including rescue medications, were sourced from each cost per pack at price to public plus value added tax (PTP + VAT), as listed in the 2017 catalogue of sanitarian products included in the Spanish National Healthcare System (Table 2) [37]. The cost per pack for UMEC/VI and TIO/OLO was €70.25 and €81.49, respectively. Costs for rescue medications were also considered within the model; based on the results of the UMEC/VI versus TIO/OLO head-to-head study [25], salbutamol 1.77 inhalations per day and 1.51 inhalations per day were modeled into the TIO/OLO and UMEC/VI arms, respectively (cost per pack €2.69; Table 2).

**Table 2:** Drug acquisition costs

Drug (brand)	Drug (generic)	Dose (mcg)	Pack size	Pack cost (PTP + VAT)^a^ [26] Pack cost (PTP + VAT)^a^ [26]	Dosing	Annual acquisition cost^a^
Comparator drug costs						
Spiolto Respimat	TIO/OLO	5/5	30 doses (60 pulsations)	€81.49	2 inhalations once daily	€992
Anoro Ellipta	UMEC/VI	62.5/25	30 doses	€70.25	1 inhalation once daily	€855
Subsequent treatment and other drugs costs						
Spiriva Handihaler^b^	TIO	18	30 doses	€49.06	1 inhalation once daily	€597
Seretide Accuhaler^b^	SAL/FP	50/500	60 doses	€41.28	1 inhalation twice daily	€503
Flixotide Accuhaler^c^	FP	500	60 doses	€31.47	2 inhalations once daily	€383
Ventolin^d^	Salbutamol	100	200 doses	€2.69	Based on head-to-head study [25]Based on head-to-head study [25]	

^a^Source: Pack cost is taken from the Ministerio de Sanidad, Servicios Sociales e Igualdad. Available at: https://www.msssi.gob.es/en/home.htm Accessed February 2018

^b^TIO + SAL/FP administered together as escalation treatment for both UMEC/VI and TIO/OLO arm in base case

^c^added on to UMEC/VI in an escalation strategy tested in a sensitivity analysis

^d^rescue medication, modeled based on data from the head-to-head study [25]: 1.77 dose inhalations per day for TIO/OLO, 1.51 dose inhalations per day for UMEC/VI

*FP* fluticasone propionate, *PTP* price to public, *SAL* salmeterol xinafoate, *TIO/OLO* tiotropium/olodaterol, *UMEC/VI* umeclidinium/vilanterol, *VAT* value added tax

The model assigned the unit cost of an exacerbation to the number of exacerbations experienced in each cycle. Cost for exacerbations and disease management were sourced from the previous cost-effectiveness analysis in Spain [26] and were inflated to 2017 Euro valuations using the Spanish Consumer Price Index [38]. The costs of a moderate and severe exacerbation were estimated to be €72.80 and €4470, respectively (Table 1) [26].

Annual disease management costs reflected the level of follow-up based on the proportion of patients with various levels of dyspnea in each model cycle (€524.87/year for a patient without dyspnea symptoms; €699.98/year for a patient experiencing dyspnea symptoms several days per week; €925.85/year for patients with dyspnea symptoms most days per week; Table 1).

#### Utilities

In this Spanish cost-effectiveness analysis, the model estimated utilities in the base case analysis via linear regression for each cycle, using a utility equation developed for the previous cost-effectiveness analysis of UMEC/VI versus TIO [26], based on data from an observational study in Spain [39]. Details of the utility estimates used within this study, including any modifications made to the Spanish risk equation from the initial disease progression model [28–32], are available in Additional file 2.

#### Model assumptions

Given the disparity between the cycle length (1 year) and the total duration of the UMEC/VI versus TIO/OLO head-to-head study (8-week treatment periods) [25], it was assumed that treatment effects started at 0 months, i.e. a delay in treatment effect was not modeled. Clinically relevant effects have been seen after the first dose of treatment in favor of UMEC/VI, in studies performing serial FEV_1_ assessment [40]. The model also assumed that treatment effects did not wane over the duration of the analysis and that the differential treatment effects observed within the UMEC/VI versus TIO/OLO head-to-head study were maintained over the time horizon, as long as patients remained on treatment. This assumption was validated by the Spanish clinical experts.

Discontinuation rates of 8.7% per year for each arm, were based on the rates observed in the Understanding Potential Long-Term Impacts on Function with Tiotropium (UPLIFT; NCT00144339) trial among GOLD stage 2 patients in the TIO arm [41]. Once patients discontinued their original treatment, the analysis assumed patients would escalate to multiple inhaler triple therapy (ICS, LAMA and LABA in combination). Spanish clinical experts confirmed the chosen escalation treatment in Spain as salbutamol/fluticasone propionate (SAL/FP) 50/500 mcg, two inhalations per day plus TIO 18 mcg, one inhalation per day. In the base case, the model assumed that the lung function of patients who escalated to triple therapy would not be subject to any lasting benefit from their initial treatment. Therefore, a + 52 mL benefit in FEV_1_ was applied for patients escalating from TIO/OLO to triple therapy (equal to the treatment difference between UMEC/VI and TIO/OLO) and no change in FEV_1_ was applied for patients escalating from UMEC/VI to triple therapy, therefore cancelling the FEV_1_ benefit conferred by the initial treatment.

### Base case settings

A time horizon of 3 years was employed to align with the previous UMEC/VI versus TIO cost-effectiveness analysis [26] and with another cost-effectiveness analysis performed on treatments for COPD [42]. Costs and benefits were discounted at 3% per year in line with Spanish guidelines for economic evaluation [43].

### Model outputs

The model estimated: exacerbation rates (number of moderate and severe exacerbations per patient per year), costs (total, drug, non-drug; discounted), survival, life-years (LY) gained (undiscounted), QALYs gained (discounted), and incremental cost effectiveness per LY and per QALY gained (i.e. the cost effectiveness of each treatment for each year of survival [LY] and for each LY adjusted for quality of life [QALY]).

### Sensitivity and scenario analyses

A scenario analysis was conducted using baseline characteristics from the ITT population from the UMEC/VI versus TIO/OLO head-to-head study (Table 1) [25]. All other parameters remained as per the base case, with the exception of utilities, which are outlined below.

In the ITT scenario analysis, utilities were estimated using the GALAXY model algorithm [28, 29], based on St. George’s Respiratory Questionnaire for COPD patients (SGRQ-C) in each model cycle. First, the pooled baseline COPD Assessment Test (CAT) in the UMEC/VI versus TIO/OLO study (mean: 17.76, standard error [SE]: 0.46) was re-scaled to match the 0–100 range of SGRQ-C, by multiplying by 2.5 (estimated SGRQ-C: 44.4). Second, SGRQ-C was converted to SGRQ using the following conversion: SGRQ = [SGRQ-C*0.9] + 3.1 = 43.1. At baseline, this provided an estimate for SGRQ value of 43.1. This baseline SGRQ value was transformed into an EuroQol-5 Dimension questionnaire (EQ-5D) utility estimate using the algorithm developed by Starkie et al. [44]: EQ-5D = 0.9617–0.0013*SGRQ total − 0.0001*SGRQ total^2^ + 0.0231*%male. The corresponding baseline EQ-5D utility score was 0.743. Utilities in subsequent cycles were calculated from the SGRQ-C scores using the same approach.

Deterministic sensitivity analyses were conducted to examine the impact of changing certain model parameters estimates and are listed in Table 3. A threshold analysis was also conducted on the price of TIO/OLO, for UMEC/VI to be cost effective at €30,000/QALY.

**Table 3:** Deterministic sensitivity analyses

Parameter	Base case	Sensitivity analysis
Time horizon	3 years	1,5 and 10 years and lifetime (25 years) time horizons
Discount rate	3%	0% and 5%
Patient population and utility estimation	Equation developed in the previous cost-effectiveness analysis [26], based on an observational Spanish study [39]	Base case population with utilities estimated from GALAXY utility algorithm ITT study population with utilities estimated from GALAXY utility algorithm ITT study population with utilities estimated from GALAXY utility algorithm over a lifetime horizon
FEV_1_ treatment effect	UMEC/VI 180 mL, TIO/OLO 128 mL (incremental FEV_1_ treatment effect of 52 mL (favoring UMEC/VI)	Equal FEV_1_ treatment effect (128 mL) for UMEC/VI and TIO/OLO Incremental FEV_1_ treatment effect with UMEC/VI equal to the upper (+ 77 mL) and lower (+ 28 mL) 95% CI
Treatment discontinuation	8.7%	50% for year 1 (from population-based, retrospective, observational study in Catalonia [55]) and 8.7% (from the UPLIFT trial [41]) for subsequent years
Subsequent treatment	SAL/FP 50/500 mcg, two inhalations per day + TIO 18 mcg	Patients on UMEC/VI add FP 500 mcg, two inhalations per day; patients on TIO/OLO escalate to SAL/FP 500/50 mcg, two inhalations per day + TIO 18 mcg, one inhalation per day^a^
Costing for dyspnea^b^	€524.87/year; €699.98/year; €925.85/year	Cost of level of dyspnea ±20%
Costing for exacerbations^b^	Moderate €72.76^c^; severe €4466.09^d^	Cost of exacerbations ±20%

*CI* confidence interval, *ED* emergency department, *FEV*_*1*_ forced expiratory volume in one second, *FP* fluticasone propionate, *ITT* intent-to-treat, *OCS* oral corticosteroid, *SAL* salmeterol xinafoate, *TIO/OLO* tiotropium/olodaterol, *UMEC/VI* umeclidinium vilanterol, *UPLIFT* Understanding Potential Long-Term Impacts on Function with Tiotropium

^a^This sensitivity analysis assumed that patients in both the UMEC/VI and TIO/OLO arms experienced the same FEV_1_ improvement upon escalating to triple therapy (assumed to be + 52 mL)

^b^inflated to 2017 Euros using the Consumer Price Index [38]

^c^cost of OCS and/or antibiotics, one primary care visit and one ED visit for 4.3% patients

^d^cost of one primary care visit, one ED visit, and hospitalization for 8 days

### Probabilistic sensitivity analysis

Probabilistic sensitivity analyses (PSA) were conducted to address the uncertainty in the model input values, by assigning distributions to input parameters and randomly sampling from these distributions over 2000 simulations. Two probabilistic analyses were conducted: one for the base case and one for the ITT scenario analysis. Discontinuation rates used beta distributions with SE equal to 20% of the point estimates. Treatment effects on FEV_1_ was assigned a normal distribution with the observed 95% CIs. Exacerbation event costs and annual health state costs used a gamma distribution with SE equal to 20% of the point estimate. Risk equation coefficients were sampled using correlated draws from a Cholesky decomposition table, obtained from the covariance matrices for each equation [28].

## Results

In the base case analysis, UMEC/VI was associated with fewer exacerbations (− 0.014 per year) and improvements in survival (+ 0.004 LYs) and quality of life (+ 0.029 QALYs) over a 3-year time horizon, when compared with TIO/OLO. Treatment with UMEC/VI also resulted in a cost saving of €393 per patient (Table 4).

**Table 4:** Model results: base case

Deterministic	TIO/OLO	UMEC/VI	Difference
Average number of exacerbations, per patient per life-year			
Severe	0.074	0.070	−0.004
Total (moderate and severe)	0.589	0.575	−0.014
Outcomes at end of 3 years			
Survival at end of time horizon	89.9%	90.3%	0.4%
Undiscounted Lys	2.870	2.874	0.004
Discounted (3% p.a.) QALY	2.118	2.147	0.029
Costs at end of 3 years			
Drug costs	€2820	€2490	−€335
Non-drug costs	€3210	€3160	−€58
Exacerbation event costs	€1020	€973	−€47
Health state costs (by dyspnea severity)	€2190	€2180	−€10
Total costs	€6040	€5640	−€393
Incremental results (versus TIO/OLO)			
ICER (€ per QALY gained)		Dominant	
ICER (€ per LY gained)		Dominant	

Cost and cost-effectiveness data are presented to three significant figures for values of four figures or more, and to the nearest Euro for values of three figures or less

*ICER* incremental cost-effectiveness ratio, *LY* life years, *QALY* quality-adjusted life-year, *p.a.* per annum, *TIO/OLO* tiotropium/olodaterol, *UMEC/VI* umeclidinium/vilanterol

The results of the ITT scenario analysis were consistent with the base case, showing that UMEC/VI was associated with fewer exacerbations (− 0.014 per year) and improvements in survival (+ 0.003 LYs) and quality of life (+ 0.009 QALY) over a 3-year time horizon, when compared with TIO/OLO, alongside cost savings of €396 per patient (Table 5).

**Table 5:** Model results: ITT scenario analysis

Deterministic	TIO/OLO	UMEC/VI	Difference
Average number of exacerbations, per patient per life-year			
Severe	0.077	0.074	−0.004
Total	0.613	0.599	−0.014
Outcomes at end of 3 years			
Survival at end of time horizon	91.1%	91.4%	0.3%
Undiscounted LYs	2.885	2.889	0.003
Discounted (3% p.a.) QALYs	2.050	2.060	0.009
Costs at end of 3 years			
Drug costs	€2840	€2500	−€337
Non-drug costs	€3300	€3240	−€58
Exacerbation event costs	€1080	€1030	−€48
Health state costs (by dyspnea)	€2220	€2210	−€10
Total costs	€6130	€5740	−€396
Incremental results (versus TIO/OLO)			
ICER (€ per QALY gained)		Dominant	
ICER (€ per LY gained)		Dominant	

Cost and cost-effectiveness data are presented to three significant figures for values of four figures or more, and to the nearest Euro for values of three figures or less

*ICER* incremental cost-effectiveness ratio, *ITT* intention-to-treat, *LY* life years, *p.a.* per annum, *QALY* quality-adjusted life-year, *TIO/OLO* tiotropium/olodaterol, *UMEC/VI* umeclidinium/vilanterol

The results of sensitivity analyses are presented in Table 6, looking at the impact of the time horizon, discount rates, patient population and utility estimation, treatment effect, discontinuation, subsequent treatment, and costings on the model outcomes. UMEC/VI was found to be dominant (better outcomes and reduced costs) compared with TIO/OLO in all analyses, with the exception of one analysis (equal FEV_1_ absolute treatment effect in both arms [128 mL]), whereby UMEC/VI was considered equally effective but less expensive (Table 6). A sensitivity analysis conducted on the price of TIO/OLO found that UMEC/VI was cost effective at a willingness-to-pay threshold of €30,000/QALY until the price of TIO/OLO fell to €39.66 (51% reduction from its current list price at €81.49).

**Table 6:** Model results: sensitivity analyses

Sensitivity analyses	Incremental costs	Incremental QALY	ICER (€ per QALY gained)
Base case	−€393	0.029	Dominant
Time horizon			
1 year	−€152	0.010	Dominant
5 years	−€562	0.043	Dominant
10 years	−€752	0.071	Dominant
Lifetime (25 years)	−€774	0.091	Dominant
Discount rate for costs and benefits			
0%	−€405	0.029	Dominant
5%	−€388	0.027	Dominant
Patient population and utility estimation			
Base case population with utilities estimated from the GALAXY utility algorithm	−€393	0.010	Dominant
Use ITT study population with utilities estimated from the GALAXY utility algorithm	−€396	0.009	Dominant
Use ITT study population with utilities estimated from the GALAXY utility algorithm with life time horizon	−€781	0.052	Dominant
Treatment effect			
Analysis with equal FEV_1_ treatment effect (128 mL) across UMEC/VI and TIO/OLO arms after initiating therapy	−€338	0.000	Equally effective, less expensive
Incremental FEV_1_ treatment effect with UMEC/VI after initiating therapy equal to the upper 95% CI (+ 77 mL)	−€420	0.041	Dominant
Incremental FEV_1_ treatment effect with UMEC/VI after initiating therapy equal to the lower 95% CI (+ 28 mL)	−€362	0.012	Dominant
Treatment discontinuation			
Treatment discontinuation rates from first year sourced from population-based, retrospective, observational study in Catalonia (50%) and from the UPLIFT trial for subsequent years (8.7%)	−€252	0.018	Dominant
Subsequent treatment			
Patients on UMEC/VI escalate to UMEC/VI and FP 500 mcg, two inhalations per day while patients on TIO/OLO escalate to SAL/FP 500/50 mcg, two inhalations per day + TIO 18 mcg, one inhalation per day	−€355	0.032	Dominant
Costing			
Cost of level of dyspnea plus 20%	−€397	0.029	Dominant
Cost of level of dyspnea minus 20%	−€392	0.029	Dominant
Cost of exacerbations plus 20%	−€404	0.029	Dominant
Cost of exacerbations minus 20%	−€385	0.029	Dominant

*CI* confidence interval, *FEV*_*1*_ forced expiratory volume in one second, *FP* fluticasone propionate, *ICER* incremental cost-effectiveness ratio, *ITT* intention-to-treat, *LY* life years, *QALY* quality-adjusted life-year; *SAL* salmeterol xinafoate, *TIO/OLO* tiotropium/olodaterol, *UMEC/VI* umeclidinium/vilanterol, *UPLIFT* Understanding Potential Long-Term Impacts on Function with Tiotropium

**Fig. 1:**
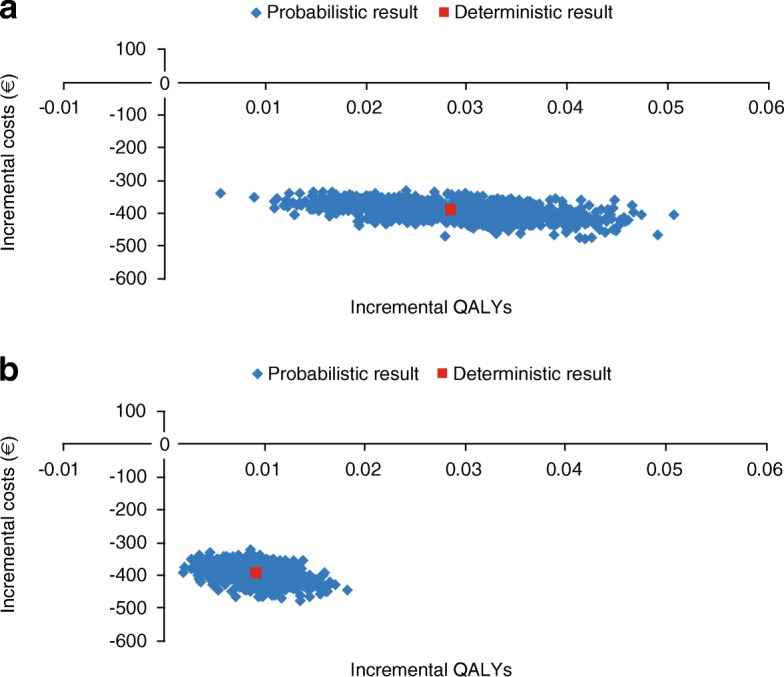
Probabilistic sensitivity analysis results for **a** base case, and **b** ITT analysis. ITT, intent-to-treat; QALY, quality-adjusted life-year

The probabilistic sensitivity analyses showed UMEC/VI to be more effective and less expensive than TIO/OLO in 100% of the simulations in the cost-effectiveness scatter plot, in both the base case and ITT scenario analysis (Fig. 1). In the base case PSA, mean costs (95% CI) for UMEC/VI and TIO/OLO were €5660 (€5000, €6410) and €6060 (€5380, €6830), respectively. Mean QALYs (95% CI) were 2.15 (2.08, 2.21) and 2.12 (2.05, 2.18) for UMEC/VI and TIO/OLO, respectively. In the ITT scenario PSA, mean costs (95% CI) for UMEC/VI and TIO/OLO were €5740 (€5060, €6490) and €6140 (€5460, €6910), respectively. Mean QALYs (95% CI) were 2.06 (1.98, 2.13) and 2.05 (1.97, 2.12) for UMEC/VI and TIO/OLO, respectively.

## Discussion

This study aimed to assess the cost effectiveness of UMEC/VI versus TIO/OLO from a Spanish National Healthcare System perspective. The results of this analysis with a Spanish population as base case, demonstrated that UMEC/VI dominated TIO/OLO, providing gains in LYs and QALYs (+ 0.004 and + 0.029, respectively) alongside total cost savings of €393 per patient over 3 years. Drug costs were the main driver of the total costs savings, followed by non-drug costs, specifically costs associated with exacerbation events. The model also showed small numerical reductions in total exacerbation rates (− 0.014 exacerbations per year) with UMEC/VI compared with TIO/OLO. The results within the ITT scenario analysis using the UMEC/VI versus TIO/OLO head-to-head study population were consistent with the base case, with UMEC/VI shown to be more effective than TIO/OLO and at a lower cost. The dominance of UMEC/VI over TIO/OLO demonstrated in both the base case and the ITT scenario analyses were maintained when a 5- and 10-year time horizon was employed, and across several sensitivity analyses. The results of the ITT utility estimation scenario analysis are particularly relevant when considering the cost effectiveness of UMEC/VI versus TIO/OLO outside of the Spanish National Healthcare System perspective. Two sensitivity analyses were conducted using the ITT population from the UMEC/VI versus TIO/OLO head-to-head study [25], with a utility estimation based on the GALAXY utility algorithm [28, 29], which converted SGRQ scores to utilities according to the algorithm of Starkie et al. [44]. Both analyses, one of which was over a 3-year period and one of which used a lifetime time horizon, demonstrated that UMEC/VI dominated TIO/OLO as the more effective and less expensive treatment. While these sensitivity analyses used Spanish costs, they would nevertheless provide the most applicable platform by which to estimate the cost effectiveness of UMEC/VI versus TIO/OLO from other national perspectives, using nationally derived cost inputs.

Although UMEC/VI was statistically superior to TIO/OLO in terms of trough FEV_1_ improvement [25], a sensitivity analysis considered the possibility of equal FEV_1_ benefit for both treatments; in this scenario, UMEC/VI was a cost-saving treatment option compared with TIO/OLO (as UMEC/VI has a lower acquisition cost). The sensitivity analyses also demonstrated that UMEC/VI QALY gains increased with the time horizon, from 0.029 QALYs in the 3-year time horizon used for the base case analysis to 0.091 QALYs in the lifetime horizon. These are relatively low QALY gains, however it should be noted that 95% of the population used within the base case had a low exacerbation risk (GOLD stage B) and high symptom burden (100% of patients had an mMRC score ≥ 2) and only 5% were classified as having a high exacerbation risk (GOLD stage D) [25]. Given the sensitivity of the model to exacerbations, this may account for the relatively low QALY gains observed.

Given that UMEC/VI has demonstrated improved efficacy when compared with TIO/OLO [25], and that UMEC/VI is less expensive than TIO/OLO in Spain, it was anticipated that this analysis would find that UMEC/VI dominated TIO/OLO as a more effective and less expensive treatment. Nevertheless, it was considered important to challenge this hypothesis using both a Spanish population as base case, and a more generalized population within a scenario analysis, in order to demonstrate the robustness of the data supporting the cost effectiveness of UMEC/VI versus TIO/OLO. While this cost effectiveness was performed from a Spanish National Healthcare System perspective, its results are generalizable to other countries, as indicated by the ITT scenario analysis, especially in relation to the health gains observed (costs are more likely to vary according to each country).

In the base case results, patients gained more QALYs than LYs, indicating that the treatment effect of UMEC/VI on FEV_1_ benefit was associated with improvement in quality of life, but little to no reduction in mortality, which is likely the result of modeling treatment effect based on FEV_1_ benefit. These results are consistent with previous studies of UMEC/VI and with systematic reviews and meta-analyses of other therapies, which have found that FEV_1_ benefit is associated with improvement in quality of life, but little to no reduction in mortality [12, 16, 45, 46]. A similar result was observed within the ITT scenario analysis; however, the magnitude of benefit was lower in this population compared with the base case, possibly due to the different approaches to utility estimation used within the base case and ITT analyses. The Spanish utility equation used within the base case, and the SGRQ-C equation used within the ITT analysis considered different variables and would therefore be sensitive to different parameters. In addition, the populations used within each equation would lead to different estimates of utility.

This study is the first to compare two LAMA/LABA dual therapies using direct head-to-head data from a randomized, 12-week study [24], which is the most reliable set of data to include within a cost-effectiveness model [24]. Other studies have compared LAMA/LABA dual therapy using models with assumptions based on network meta-analyses [47, 48], or have used head-to-head data to compare UMEC/VI with TIO monotherapy [26, 49]. For example, a UK cost-effectiveness analysis of TIO/OLO found that costs and QALYs associated with TIO/OLO were equal to those of UMEC/VI and IND/GLY. However, this study was based on indirect data from a network meta-analysis and assumed equal efficacy and equal acquisition costs for LAMA/LABA comparators [48]. Similarly, a cost-effectiveness analysis conducted in Spain used efficacy inputs derived from a meta-analysis and found that aclidinium/formoterol (ACL/FF) 400/12 mcg provided the same health benefits as TIO/OLO in terms of LYs (4.073) and QALYs (2.928), but that ACL/FF was associated with lower costs (−€332) over a 5-year time horizon [47]. An additional cost-effectiveness analysis conducted in the US found UMEC/VI to be equally effective but less costly compared with open dual LAMA/LABA combination, with efficacy inputs derived from two clinical studies [14, 16, 49].

Several cost-effectiveness analyses have been conducted from a Spanish National Healthcare System perspective, using direct head-to-head data. One study compared UMEC and TIO [50], using direct head-to-head data from a 12-week study whereby in which UMEC was found to be superior to TIO for trough FEV_1_ in the ITT population (treatment difference: 53 mL, 95% CI: 25, 81 mL; *p* < 0.001) [24]. Results from this cost-effectiveness analysis using direct head-to-head data from the UMEC and TIO study showed results comparable to our study, whereby UMEC dominated TIO, gaining similar 0.014 QALYs and demonstrating cost savings of €192 [50]. Another Spanish cost-effectiveness analysis of UMEC/VI versus TIO alone estimated 3-year costs of €6215 and QALYs of 2.025 for patients treated with UMEC/VI and found UMEC/VI to be more effective and costlier than TIO monotherapy, with an incremental cost-effectiveness ratio (ICER) of €21,475/QALY [26]. The higher QALYs and lower costs for UMEC/VI in our base case analysis reflect the patient population characteristics at baseline. The Spanish population in our analysis had fewer current smokers, was less obese, had a lower cardiovascular disease comorbidity, experienced fewer exacerbations and had a higher FEV_1_% predicted at baseline compared with the population in the Spanish cost-effectiveness analysis of UMEC/VI versus TIO [26].

The cost effectiveness analysis presented here could be a conservative estimate, as some medical costs such as costs associated with inhaler misuse, were not taken into account within the model [51, 52]. Poor inhalation technique can lead to poor disease control and the costs associated with critical errors are considerable [51, 52]. In Spain, total costs associated with poor inhalation technique were estimated at €155 million in 2015, representing 15.5% of the total costs associated with unscheduled healthcare visits [51]. A recent study compared the Ellipta device with other commonly used inhaler devices, and the results demonstrated that fewer patients had at least one critical error using the Ellipta device compared with five alternative inhalers [53]. In addition, the Ellipta inhaler received more positive patient feedback in terms of ease of use in the UMEC/VI versus TIO/OLO head-to-head study, compared with the Respimat inhaler [25]. Inhaler training was provided in the UMEC/VI versus TIO/OLO head-to-head study, and inhaler technique was routinely checked, thus reiterating how the results of this cost-effectiveness analysis are likely to be a conservative estimate of treatment difference, as they do not fully reflect the development of poor technique over time and variation in inhaler training seen in a real-world setting.

The results of this study are based on data from the 8-week UMEC/VI versus TIO/OLO head-to-head study [25]. Future studies to confirm the results observed within this direct treatment comparison, and to compare the cost effectiveness of LAMA/LABA dual therapies over a longer duration, would be beneficial in further helping payers make informed judgements on the cost effectiveness of LAMA/LABA therapies.

As there are currently no clinical studies providing data on the use of LAMA/LABA combination therapy for a longer duration than the timeframes considered within this analysis (3 years, 5 years, 10 years and lifetime), certain assumptions were made within this model. One such assumption was that treatment effects began at the outset of the analysis (month 0) in both arms. This assumption is supported by data from studies that demonstrated observable, significant improvement in FEV_1_ from the first dose [12, 40]. The model also assumed that treatment effect remained constant for the duration of the study, as long as patients remained on treatment. Treatment effect differences between UMEC/VI and TIO/OLO may be driven by the differences seen between the LAMA components UMEC and TIO. A randomized, blinded, 12-week parallel group study demonstrated superiority of UMEC over TIO on the primary endpoint of trough FEV_1_ [24], and the Understanding Potential Long-Term Impacts on Function with Tiotropium (UPLIFT) study showed that LAMA treatment effects on FEV_1_ and health status do not wane over time (at least up to 4 years) [54]. This was shown in both the overall population [54] and the subgroup of patients with GOLD stage II COPD [41], as included in the analysis presented here. An additional study demonstrated significant improvement in FEV_1_ for patients treated with UMEC/VI, and this treatment benefit was maintained over 6 months [12]. Based on these previous studies, it was therefore reasonable to assume that the differential treatment effect seen between UMEC/VI and TIO/OLO would be maintained over a 3-year time period [24, 54].

Discontinuation rates used within the base case were based on data from the UPLIFT study [41], as it provides data over a 4-year follow-up, and this approach was validated by Spanish clinical experts [41]. Nevertheless, a sensitivity analysis was conducted using 50% discontinuation rates sourced from an observational study in Catalonia [55], and UMEC/VI remained dominant compared with TIO/OLO. Data on escalation to triple therapy from LAMA/LABA therapy are also not available from previous clinical studies, therefore it was necessary to make an assumption regarding the changes in FEV_1_ once a patient escalated to subsequent triple therapy (LAMA/LABA+ICS). The base case assumed that patients would escalate to the same triple therapy regimen, and that FEV_1_ treatment effect once on triple therapy would be the same between arms. This may have been a conservative assumption as it assumed that patients treated with UMEC/VI did not experience a change in FEV_1_ after they initiated ICS therapy. Indeed, this assumption was tested in a sensitivity analysis and an increase in QALY gains of 0.004 versus the base case was observed. Finally, data on baseline fibrinogen concentration and 6MWT distances were not available from the sources used for this analysis and had to be estimated within the model. It may be informative to collect and include such data within future studies to assist further cost-effectiveness evaluations.

## Conclusions

This analysis has shown UMEC/VI to be both more effective and less expensive than TIO/OLO for patients with symptomatic COPD in Spain, providing small additional gains in LY (0.004) and QALY (0.029), as well as a cost saving of €393 per patient over a 3-year time period. Scenario and sensitivity analyses demonstrated results consistent with the base case. These data may aid payers in making judgements on which LAMA/LABA treatments can be considered cost effective in a Spanish setting.

## Additional files

Additional file 1: Improvement in COPD disease factors in the model at time = 0. Description of the improvements in COPD disease factors built into the model at time 0 (i.e. the beginning of the cycle), when FEV_1_ benefit has been included. (DOCX 13 kb)

Additional file 2: Utilities. Description of the utilities used within the study, including any modifications. (DOCX 13 kb)

## Abbreviations

6MWT: 6-min walk test
CAT: COPD Assessment Test
CI: Confidence interval
COPD: Chronic obstructive pulmonary disease
ECLIPSE: Evaluation of COPD Longitudinally to Identify Predictive Surrogate Endpoints
EMA: European Medicines Agency
EQ 5D: EuroQol-5 Dimension questionnaire
FEV1: Forced expiratory volume in 1 s
FP: Fluticasone propionate
GesEPOC: Guía española de la enfermedad pulmonar obstructiva crónica
GOLD: Global Initiative for Chronic Obstructive Lung Disease
ICS: Inhaled corticosteroid
ITT: Intent to-treat
LABA: Long-acting β2-agonist
LAMA: Long-acting muscarinic antagonist
LY: Life-years
mMRC: Modified Medical Research Council
OLO: Olodaterol
PP: Per-protocol
PSA: Probabilistic sensitivity analyses
PTP: Price to public
QALYs: Quality-adjusted life years
SAL: Salbutamol
SE: Standard error
SGRQ-C: St. George’s Respiratory Questionnaire for COPD patients
TIO: Tiotropium
UMEC: Umeclidinium bromide
UPLIFT: Understanding Potential Long-Term Impacts on Function with Tiotropium
VAT: Value added tax
VI: Vilanterol
