# Correction to: Cost-effectiveness analysis of umeclidinium bromide/vilanterol 62.5/25 mcg versus tiotropium/olodaterol 5/5 mcg in symptomatic patients with chronic obstructive pulmonary disease: a Spanish National Healthcare System perspective

**DOI:** 10.1186/s12931-019-0985-2

**Published:** 2019-01-28

**Authors:** M. T. Driessen, J. Whalen, B. Seewoodharry Buguth, L. A. Vallejo-Aparicio, I. P. Naya, Y. Asukai, B. Alcázar-Navarrete, M. Miravitlles, F. García-Río, N. A. Risebrough

**Affiliations:** 1 Value Evidence and Outcomes, GSK, 980 Great West Road, Brentford, Middlesex TW8 9GS UK; 2 ICON Health Economics, ICON plc, Abingdon, UK; 3 Departamento de Evaluación de Medicamentos, GSK, Tres Cantos, Madrid, Spain; 4 Global Respiratory Franchise, GSK, Brentford, Middlesex UK; 5 Hospital de Alta Resolución de Loja, Granada, Spain; 6 Pneumology Department, Hospital Universitari Vall d’Hebron, Barcelona, Spain; 7 Hospital Universitario La Paz-IdiPAZ, Madrid, Spain; 8 ICON Health Economics, ICON, Toronto, ON Canada

**Correction to: Respir Res**

**https://doi.org/10.1186/s12931-018-0916-7**

Following publication of the original article [1], we have been notified of the below corrections to the 5th paragraph in the Discussion section.

- Reference 24 needs to be amended to 25
- The 12-week study needs to be corrected to 8-week study

Corrected text:

This study is the first to compare two LAMA/LABA dual therapies using direct head-to-head data from a randomized, 8-week study [25], which is the most reliable set of data to include within a cost-effectiveness model [25].
