# Supplementary material for: Cost-effectiveness analysis of umeclidinium bromide/vilanterol 62.5/25 mcg versus tiotropium/olodaterol 5/5 mcg in symptomatic patients with chronic obstructive pulmonary disease: a Spanish National Healthcare System perspective
Source: Respir Res. 2018 Nov 20;19:224. doi: 10.1186/s12931-018-0916-7 (PMC6245710; doi:10.1186/s12931-018-0916-7)
Supplement: Supplementary file 1 — Improvement in COPD disease factors in the model at time = 0. Description of the improvements in COPD disease factors built into the model at time 0 (i.e. the beginning of the cycle), when FEV1 benefit has been included. (DOCX 13 kb) [file 12931_2018_916_MOESM1_ESM.docx]

**Additional File 1. Improvement in COPD disease factors in the model at time = 0.**

Description of the improvements in COPD disease factors built into the model at time 0 (i.e. the beginning of the cycle), when FEV_1_ benefit has been included.

| **Treatment** | **Starting FEV_1_ (mL)** | **FEV_1_ absolute treatment effect (mL)** | **FEV % predicted** | **Dyspnea (no days)** | **Dyspnea (several days)** | **Dyspnea (most days)** | **Cough/sputum** | **6MWT distance (m)** | **SGRQ-C** |
| --- | --- | --- | --- | --- | --- | --- | --- | --- | --- |
| **UMEC/VI** | 1563 | +180 | +6.8% | +1.1% | +5.5% | −6.6% | −0.9% | +10.8 | −1.9 |
| **TIO/OLO** | 1563 | +128 | +4.8% | +0.7% | +3.8% | −4.5% | −0.6% | +8.1 | −1.6 |

COPD, chronic obstructive pulmonary disease; FEV_1_, forced expiratory volume in one second; SGRQ-C, St. George’s respiratory questionnaire for COPD patients;
TIO/OLO, tiotropium/olodaterol; UMEC/VI, umeclidinium/vilanterol; 6MWT, 6-minute walking test.
