# Supplementary material for: Cost-effectiveness analysis of umeclidinium bromide/vilanterol 62.5/25 mcg versus tiotropium/olodaterol 5/5 mcg in symptomatic patients with chronic obstructive pulmonary disease: a Spanish National Healthcare System perspective
Source: Respir Res. 2018 Nov 20;19:224. doi: 10.1186/s12931-018-0916-7 (PMC6245710; doi:10.1186/s12931-018-0916-7)
Supplement: Supplementary file 2 — Utilities. Description of the utilities used within the study, including any modifications. (DOCX 13 kb) [file 12931_2018_916_MOESM2_ESM.docx]

**Additional File 2. Utilities.**

Description of the utilities used within the study, including any modifications.

In the GALAXY model, quality of life was assessed using an adjusted risk equation based on St. George’s respiratory questionnaire (SGRQ) scores reported in the ECLIPSE study [29], which were converted to utilities using the algorithm of Starkie et al [44]. For better adaptation to the Spanish environment, this cost-effectiveness analysis used a utility equation developed in a previous cost-effectiveness analysis of umeclidinium/vilanterol (UMEC/VI) versus tiotropium (TIO) in Spain [26]. This new risk equation estimated utility values based on data from an observational study in Spanish patients with COPD [39]. In order to do this, two modifications were made to the initial GALAXY disease progression model to implement the utility equation used within this analysis: first, as the categorical distribution of modified Medical Research Council (mMRC) scores is not estimated in the GALAXY model, a mMRC score of 2–3 was assumed equal to the modelled value for “dyspnea several days per week” and a mMRC score of 4 was assumed equal to the modelled value “dyspnea most days of the week”. The second modification was the addition of a calibration factor to increase the estimated utility. Without the calibration factor, the Spanish risk equation estimated a utility of 0.5561 at baseline; however, this utility would be very low given the baseline characteristics and symptom burden of the patient population. Another study analyzing generic utilities for Spanish patients with COPD estimated a mean utility of 0.80 for patients with moderate COPD (GOLD B) [39], and the baseline estimate for utility in the UMEC/VI versus TIO/olodaterol (OLO) head-to-head study was 0.7425 [25, 29]. Therefore, the calibration factor was implemented as the difference between the baseline utility estimate in the UMEC/VI versus TIO/OLO head-to-head study (0.7425) and the Spanish risk utility equation at baseline (0.5561) [44].
